# A Nomogram-Based Prognostic Model for Advanced Hepatocellular Carcinoma Patients Treated with Sorafenib: A Multicenter Study

**DOI:** 10.3390/cancers13112677

**Published:** 2021-05-29

**Authors:** Giovanni Marasco, Francesco Poggioli, Antonio Colecchia, Giuseppe Cabibbo, Filippo Pelizzaro, Edoardo Giovanni Giannini, Sara Marinelli, Gian Ludovico Rapaccini, Eugenio Caturelli, Mariella Di Marco, Elisabetta Biasini, Fabio Marra, Filomena Morisco, Francesco Giuseppe Foschi, Marco Zoli, Antonio Gasbarrini, Gianluca Svegliati Baroni, Alberto Masotto, Rodolfo Sacco, Giovanni Raimondo, Francesco Azzaroli, Andrea Mega, Gianpaolo Vidili, Maurizia Rossana Brunetto, Gerardo Nardone, Luigina Vanessa Alemanni, Elton Dajti, Federico Ravaioli, Davide Festi, Franco Trevisani, on behalf of the Italian Liver Cancer (ITA.LI.CA.) Group

**Affiliations:** 1 Division of Internal Medicine and Digestive Pathophysiology, IRCCS Azienda Ospedaliero-Universitaria di Bologna, 40138 Bologna, Italy; 2 Department of Medical and Surgical Science, University of Bologna, 40138 Bologna, Italy; francesco.poggioli@studio.unibo.it (F.P.); marco.zoli@unibo.it (M.Z.); francesco.azzaroli@unibo.it (F.A.); luigina.alemanni@studio.unibo.it (L.V.A.); elton.dajti2@unibo.it (E.D.); f.ravaioli@unibo.it (F.R.); davide.festi@unibo.it (D.F.); franco.trevisani@unibo.it (F.T.); 3 Gastroenterology Unit, Borgo Trento University Hospital Verona, 37126 Verona, Italy; antonio.colecchia@aovr.veneto.it; 4 Gastroenterology & Hepatology Unit, Department of Health Promotion, Mother & Child Care, Internal Medicine & Medical Specialties, PROMISE, University of Palermo, 90133 Palermo, Italy; giuseppe.cabibbo78@gmail.com; 5 Gastroenterology Unit, Department of Surgery, Oncology and Gastroenterology, University of Padua, 35124 Padua, Italy; filippo.pelizzaro@unipd.it; 6 Gastroenterology Unit, Department of Internal Medicine, IRCCS Ospedale Policlinico San Martino, 16132 Genoa, Italy; egiannini@unige.it; 7 Division of Internal Medicine, Hepatobiliary and Immunoallergologic Diseases, IRCCS Azienda Ospedaliero-Universitaria di Bologna, 40138 Bologna, Italy; sara.marinelli@aosp.bo.it; 8 Division of Internal Medicine and Gastroenterology, Complesso Integrato Columbus, Università Cattolica del Sacro Cuore, 00168 Rome, Italy; gianludovico.rapaccini@unicatt.it; 9 Gastroenterology Unit, Ospedale Belcolle, 01100 Viterbo, Italy; e.caturelli@tiscalinet.it; 10 Division of Medicine, Bolognini Hospital, 24068 Seriate, Italy; mariella.dimarco@asst-bergamoest.it; 11 Unit of Infectious Diseases and Hepatology, Azienda Ospedaliero-Universitaria di Parma, 43126 Parma, Italy; ebiasini@ao.pr.it; 12 Internal Medicine and Hepatology Unit, Department of Experimental and Clinical Medicine, University of Firenze, 50139 Florence, Italy; fabio.marra@unifi.it; 13 Gastroenterology Unit, Department of Clinical Medicine and Surgery, University of Napoli “Federico II”, 80138 Napoli, Italy; filomena.morisco@unina.it; 14 Department of Internal Medicine, Ospedale per gli Infermi di Faenza, 48018 Faenza, Italy; francesco.foschi@auslromagna.it; 15 Division of Internal Medicine, Neurovascular and Hepatometabolic Diseases, IRCCS Azienda Ospedaliero-Universitaria di Bologna, 40138 Bologna, Italy; 16 Division of Internal Medicine and Gastroenterology, Policlinico Gemelli, Università Cattolica del Sacro Cuore, 00168 Rome, Italy; antonio.gasbarrini@unicatt.it; 17 Liver Injury and Transplant Unit, Polytechnic University of Marche, 60020 Ancona, Italy; g.svegliati@univpm.it; 18 Gastroenterology Unit, IRCCS Ospedale Sacro Cuore Don Calabria, Negrar, 37024 Verona, Italy; alberto.masotto@sacrocuore.it; 19 Gastroenterology and Digestive Endoscopy Unit, Foggia University Hospital, 71100 Foggia, Italy; r.sacco@ao-pisa.toscana.it; 20 Division of Clinical and Molecular Hepatology, University of Messina, 98124 Messina, Italy; raimondo@unime.it; 21 Division of Gastroenterology, IRCCS Azienda Ospedaliero-Universitaria di Bologna, 40138 Bologna, Italy; 22 Division of Gastroenterology, Bolzano Regional Hospital, 39100 Bolzano, Italy; andrea.mega@sabes.it; 23 U.O.C. Clinica Medica, Department of Medical, Surgical and Experimental Sciences, University of Sassari, Azienda Ospedaliero-Universitaria di Sassari, 07100 Sassari, Italy; gianpaolovidili@uniss.it; 24 Hepatology and Liver Physiopathology Laboratory and Internal Medicine, Department of Clinical and Experimental Medicine, University of Pisa, 56124 Pisa, Italy; maurizia.brunetto@unipi.it; 25 Hepato-Gastroenterology Unit, Department of Clinical Medicine and Surgery, University of Naples “Federico II”, 80138 Naples, Italy; nardone@unina.it; 26 Division of Semeiotics, Department of Medical and Surgical Sciences, University of Bologna, 40138 Bologna, Italy

**Keywords:** sorafenib, hepatocellular carcinoma, survival, prognosis, cohort study

## Abstract

**Simple Summary:**

Accurate prognostic systems capable of predicting the survival of patients with advanced hepatocellular carcinoma undergoing Sorafenib therapy are still lacking. The search for the ideal predictive tool for survival and drug response is justified by the recent availability of several other drugs effective for these patients, licensed as first- and second-line treatment, other than reducing adverse events and costs. In this study, we aimed to identify simple demographic and clinical parameters able to predict survival and Sorafenib response in a large multicenter cohort. In this study, we showed that patient’s general status, liver function and damage laboratory parameters and HCC aggressiveness were associated with the outcome of Sorafenib therapy. Two predictive nomograms, helping clinicians in the therapeutic choice, were additionally created.

**Abstract:**

Among scores and staging systems used for HCC, none showed a good prognostic ability in patients with advanced HCC treated with Sorafenib. We aimed to evaluate predictive factors of overall survival (OS) and drug response in HCC patients undergoing Sorafenib included in the Italian Liver Cancer (ITA.LI.CA.) multicenter cohort. Patients in the ITA.LI.CA database treated with Sorafenib and updated on 30 June 2019 were included. Demographic and clinical data before starting Sorafenib treatment were considered. For the evaluation of predictive factors for OS, a time-dependent Cox proportional hazard model was used. A total of 1107 patients were included in our analysis. The mean age was 64.3 years and 81.7% were male. Most patients were staged as BCLC B (205, 18.9%) or C (706, 65.1%). The median time of Sorafenib administration was 4 months (interquartile range (IQR) 2–12), and the median OS was 10 months (IQR: 4–20). A total of 263 patients (33.8%) out of 780 with available evaluation experienced objective tumoral response to Sorafenib. The Eastern Cooperative Oncology Group (ECOG) Performance Status (PS) (hazard ratio (HR) 1.284), maximum tumoral diameter (HR 1.100), plasma total bilirubin (HR 1.119), aspartate amino transferase assessed as multiple of the upper normal value (HR 1.032), alpha-fetoprotein ≥200 ng/mL (HR 1.342), hemoglobin (HR 0.903) and platelet count (HR 1.002) were associated with OS at multivariate Cox regression analysis. Drug response was predicted by maximum tumoral diameter and platelet count. A novel prognostic nomogram for patients undergoing Sorafenib is hereby proposed. The novelty introduced is the comprehensive patient’s assessment using common markers of patient’s general status, liver damage and function and HCC biology. Further studies are required to test its accuracy and provide external validation.

## 1. Introduction

Sorafenib is a tyrosine-kinase inhibitor able to improve survival in patients with advanced hepatocellular carcinoma (HCC) [1]. To date, it is considered, together with Lenvatinib [2], the gold standard treatment for patients with advanced HCC not suitable for resection or locoregional treatments [3], although the combination of Atezolizumab plus Bevacivumab was proven to be superior to Sorafenib in a recent randomized controlled trial [2,4]. Second-line drugs for patients experiencing failure or toxicity of Sorafenib therapy are Regorafenib [4] Cabozantinib [5], Nivolumab [6] and Ramucirumab [7]. Since the approval of Sorafenib’s use in clinical practice, given its cost, toxicity and the variability in survival benefit [8,9,10], several studies have been carried out to identify which patients really benefit from this therapy and which should be shifted to second-line therapies or palliative care [11]. Indeed, several clinical and laboratory factors, such as tumor etiology, Child–Pugh score, alpha-fetoprotein (AFP), platelets and gamma-glutamyl transferase have been proposed as predictors of tumor progression and survival in patients on Sorafenib [12]. In addition, several prognostic scores have been specifically set up in order to evaluate the prognosis of these patients, such as the PROSASH model, its “optimized” version PROSASH-II [12,13] and the SAP score [14]. Besides, even “not dedicated” prognostic scores already used for HCC staging, such as the Barcelona Clinic Liver Cancer (BCLC), the Okuda score and the Cancer of the Liver Italian Program (CLIP) score [15,16], have been tested with the same purpose. The Italian Liver Cancer group (ITA.LI.CA) recently proposed an internally and externally validated prognostic model for patients with HCC [17,18], but even this model was derived from a population including only a small subset of patients treated with Sorafenib, suggesting that its accuracy in this subgroup still requires a validation. As a matter of fact, we recently observed that, among several prognostic models for HCC tested within the ITA.LI.CA cohort, the CLIP score showed the highest accuracy in predicting the overall survival (OS) of patients on Sorafenib, although its performance remained suboptimal (C-index 0.604) [19]. Thus, it can be said that no validated model capable of accurately evaluating the prognosis of patients on Sorafenib is currently available [11,16]. The present study aimed at identifying predictive factors of OS and tumor response in HCC patients undergoing Sorafenib.

## 2. Materials and Methods

### 2.1. ITA.LI.CA Database

The ITA.LI.CA database [8,20] contains data on 9436 HCC patients prospectively enrolled consecutively from 1 January 1987 in several primary and tertiary Italian medical centers. These data were collected prospectively and updated every 2 years. The last update was completed on 30 June 2019. The consistency of the data entry by each center is verified by the coordinator (FT). Patients gave their written informed consent to the data collection and regarding any proposed treatment, according to the Italian law. Patient data were anonymously recorded and de-identified before analysis. All ITA.LI.CA studies are conducted in accordance with the Helsinki Declaration and rely on retrospective analyses of prospectively collected data. The ethical committee of each participating center approved the creation of the ITA.LI.CA registry and its use for scientific research.

### 2.2. Design

All the patients in the ITA.LI.CA database who underwent treatment with Sorafenib and were updated up to 30 June 2019 were included. Demographic, biochemical and clinical data (i.e., etiology, Child–Pugh score, model for end-stage liver disease (MELD) score, BCLC staging system, extra-hepatic extension, maximum tumor diameter, nodule number, macrovascular invasion, AFP, and main laboratory variables) assessed before Sorafenib starting were considered. Liver tests are reported according to the ITA.LI.CA database as multiple of the upper normal limit (UNL) to standardize values among centers. The primary end point was the evaluation of OS, defined as the time elapsed between the beginning of Sorafenib treatment and death or the last follow-up visit until 30 June 2019. The secondary end point was the evaluation of tumor response to Sorafenib, measured by mRECIST criteria [21]. The time of Sorafenib administration was calculated as the time elapsed from the first and the last dose of the drug. The patient population included in the present study has been partially included in a previous analysis [19].

### 2.3. Statistical Analysis

Categorical variables were reported as absolute number and percentage, and continuous variables as mean and standard deviation (SD). Due to heterogeneity in the follow-up, time data have been reported as median and inter-quartile range (IQR). We used a multiple imputation method for handling missing data [22], which accounted for <5% for each analyzed variable. In order to assess the association with end points, a univariate analysis and a subsequent multivariate model including demographic, biochemical and clinical parameters were used. A time-dependent Cox proportional hazard model was used to detect the predictive factors of OS. We included in a backward multivariate analysis the variables showing a significant association with the event with a *p* value <0.1 at the univariate analysis, avoiding collinearity between variables. Hazard ratio (HR) and 95% confidence interval (95% CI) were calculated. For the assessment of response to Sorafenib treatment, univariate and multivariate logistic regressions were performed due to the unavailability of standardized time data for this end point; odds ratio (OR) and 95% CI were calculated. For a rapid clinical use, results of multivariate analyses were graphically translated into nomograms for both end points. For each nomogram, the following evaluations were reported: (a) discrimination (concordance index, namely area under the Receiver Operating Characteristic (ROC) curve or Harrell’s C-index); (b) calibration plot analysis; (c) decision curve analysis. We considered statistically significant a two-tailed *p* < 0.05. Statistical analysis was performed with STATA 13.0 (StataCorp LP, College Station, TX, USA).

## 3. Results

**Figure 1:**
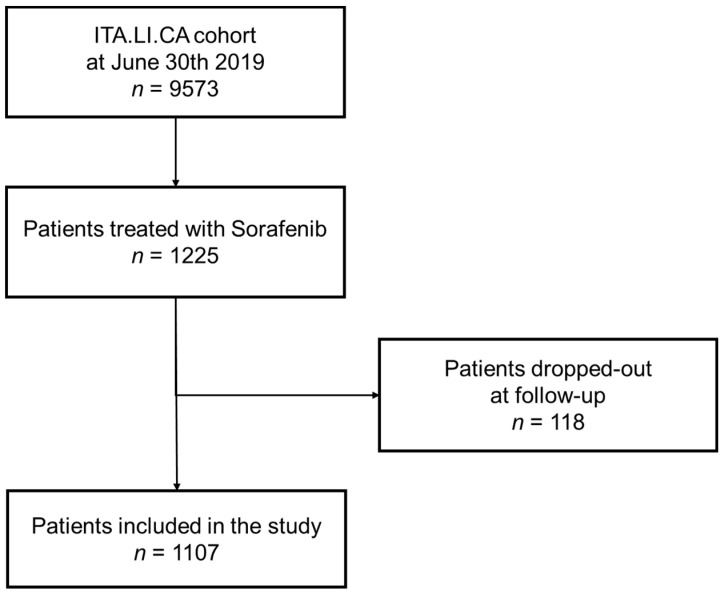
Flow-chart of the selection of ITA.LI.CA patients included in the study.

From the 9573 patients included in ITA.LI.CA database on 30 June 2019, we excluded 8348 patients who did not undergo Sorafenib treatment. After excluding 118 patients who dropped out at follow-up, we enrolled in this study 1107 patients (11.6% of the whole ITA.LI.CA cohort) (Figure 1).

### 3.1. Baseline Characteristics

The baseline characteristics of the 1107 enrolled patients are reported in Table 1. Most of them were male (81.7%) and the mean age was 64.3 years (SD: 13). Hepatitis C virus infection was the main etiological factor of the underlying liver disease (*n*. 455, 41.5%), followed by hepatitis B virus infection (10.1%), alcohol (11.5%) and metabolic disorders (6.2%). Almost two-thirds of patients belonged to Child–Pugh class A (65.6%), followed by those classified as Child–Pugh B (32.7%) and very few as C (1.7%). The mean MELD score was 10 (SD: 3.22). Patients’ general status was preserved for most patients as testified by a mean Karnofsky score of 83.0 (SD: 13.7) and an Eastern Cooperative Oncology Group (ECOG) Performance Status (PS) of 0 or 1 (56.7% and 33.7%, respectively). 

Regarding HCC characteristics, 205 (18.9%) patients were staged as BCLC B and 706 (65.1%) as BCLC C. The mean number of HCC nodules was three (SD: 2), and the maximum tumor diameter (MTD) was 4.7 cm (SD: 3.14). At Sorafenib initiation, 38.6% of the patients had metastases and 38.4% had macrovascular invasion. In 13.1% of cases, tumor extent was more than 50% of the liver volume. The mean AFP level was 2404 ng/mL (SD: 5955). During the follow-up, 882 (79.7%) patients died. The median OS was 10 months (IQR: 4–20). The median administration time of Sorafenib was 4 months (IQR: 2–12). 

Among the 780 patients with an available mRECIST evaluation, 263 (33.8%) patients experienced a tumoral response and, in particular, an objective (complete or partial) response was observed in 97 patients (12.5%) and a stable disease in 166 (21.3%). Therefore, HCC progression was reported in 517 patients (66.2%).

### 3.2. Predictive Factors of Overall Survival

Several variables were significantly associated with OS at univariate analysis. At multivariate Cox regression analysis, seven of them resisted as independent predictors of OS (Table 2):

- ECOG PS (HR, 1.284; 95% CI, 1.123–1.460; *p* < 0.001);
- MTD (HR, 1.100; 95% CI, 1.069–1.133; *p* < 0.001);
- Bilirubin (HR, 1.119; 95% CI, 1.004–1.246; *p* = 0.042);
- Multiple of AST UNL (HR, 1.032; 95% CI, 1.001–1.065; *p* = 0.041);
- AFP ≥ 200 ng/mL (HR, 1.342; 95% CI, 1.113–1.618; *p* = 0.002);
- Hemoglobin (HR, 0.903; 95% CI, 0.860–0.948; *p* < 0.001);
- Platelet count (HR, 1.002; 95% CI, 1.001–1.003; *p* < 0.001).

**Figure 2:**
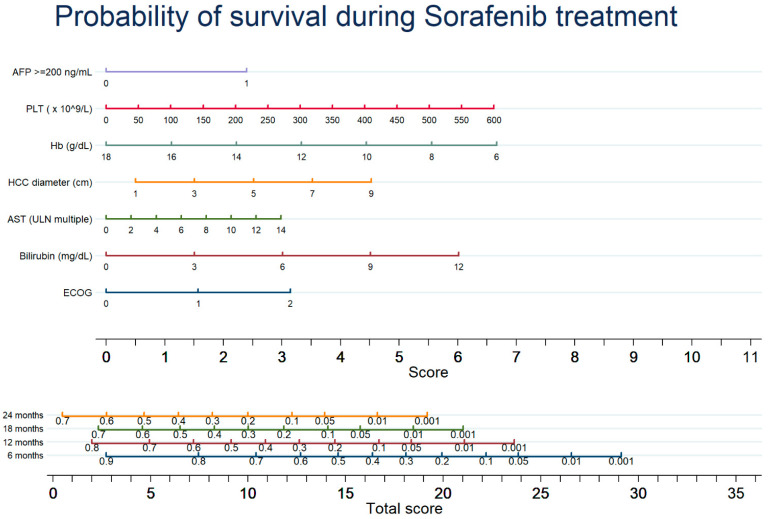
Nomogram for prediction of survival at 12, 24 and 36 months in patients with advanced HCC starting Sorafenib treatment. The effect of each variable on survival is obtained drawing a vertical line from variable’s line up to the “Score” line. The total score is obtained by adding the score for each variable. The probability of survival at each time point is obtained drawing a vertical line from “Total score” up to time point lines.

The combination of these factors for the OS prediction was graphically reported into a nomogram, stratifying OS at 6, 12, 18 and 24 months after Sorafenib start (Figure 2). The nomogram had the following discrimination ability: Harrell’s C index 0.650, Akaike Information Criterion 6885, Bayesian information criterion 6917. Calibration in predicting the 6-month OS probability and decision curves for the nomogram predicting the 6-month OS are reported in Figures S1 and S2, showing a net benefit of the nomogram use.

### 3.3. Predictive Factors of Tumor Response

**Figure 3:**
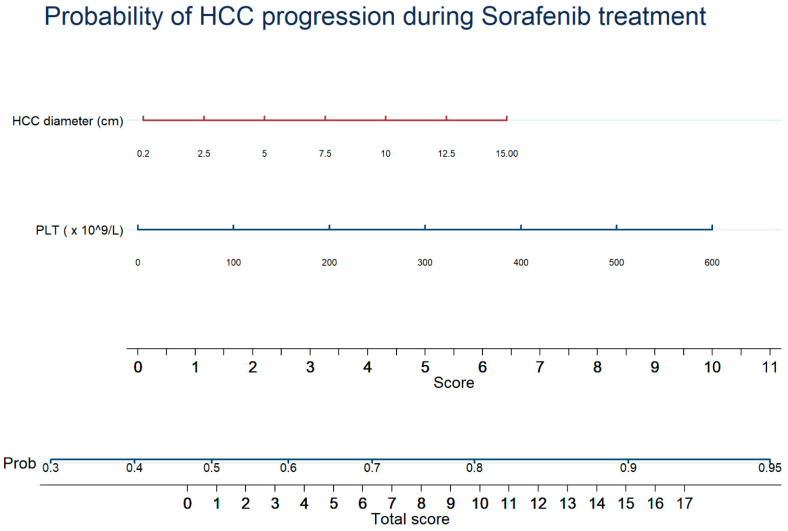
Nomogram for the prediction of radiological progression according to mRECIST criteria for patients with advanced HCC starting Sorafenib treatment. The effect of each variable on the prediction of HCC radiological progression is obtained drawing a vertical line from variable’s line up to the “Score” line. The total score is obtained by adding the score for each variable. The probability of HCC radiological progression is obtained drawing a vertical line from “Total score” up to the Prob. (probability) line.

At multivariate analysis, only two variables resisted as predictors of tumor response to Sorafenib, i.e., MTD (OR, 1.068; 95% CI, 1.006–1.134; *p* = 0.031) and platelet count (OR, 1.003; 95% CI, 1.001–1.005; *p* = 0.023) (Table 3). The combination of these two factors for the prediction of tumor response to Sorafenib is graphically reported into a nomogram (Figure 3). The area under the ROC curve for the multivariate logistic-derived model was 0.581. Calibration and decision curve analysis for the model are reported in Figures S3 and S4, showing a narrow range of net benefit.

## 4. Discussion

The present study identified the predictive factors of OS and tumor response, using ordinary variables easy to collect, in a field-practice large cohort of HCC patients undergoing Sorafenib and prospectively enrolled. The main results of the study are as follows. First, we obtained a comprehensive predictive model for OS that includes factors related to HCC burden (MTD) and aggressiveness (AFP and platelet count, the latter as a surrogate marker of platelet-derived promoting factors), liver damage and function (AST and bilirubin) and patient’s general status (ECOG PS and hemoglobin level). Second, we built a simple model to predict response to Sorafenib therapy. For both end points, we provided a graphic translation of these models through nomograms for a rapid clinical use. Based on these nomograms, as an example, a patient with an ECOP PS of 1 (1.5 points), a platelet count of 135 × 10^9^/L (1.5 points), Hb 10 g/dL (4.5 points), AFP <200 ng/mL (0 points), AST 2 UNL (0.5 point), a total bilirubin 3 mg/dL (1.5 points) and a MTD of 4 cm (2 points) totals a score of 11.5 points, corresponding to a survival probability of 65% at 6 months, 38% at 12 months, 23% at 18 months and 14% at 24 months. In parallel, referring to the predictive model of tumor response, the same patient totals a score of 3.5 points, corresponding to a probability of HCC progression over time of about 60%. Patients with advanced HCC modestly benefit from Sorafenib treatment and, also considering its toxicity and cost, the real cost-effectiveness of this therapy has been questioned [23]. The poor OS of the patients undergoing Sorafenib was confirmed by our study, showing a median value of 10.1 months, which is comparable to that of the SHARP trial [1] and better than that of the Asia-Pacific trial [24]. Notably, as the non-interventional field-practice GIDEON study [25], our investigation included patients belonging to Child–Pugh class B (33%) and BCLC stages other than C (≈30%), confirming that Sorafenib is used in clinical practice even in suboptimal candidates as well as in intermediate and early HCC cases not amenable or not responding to locoregional treatments. Several studies [12,13,14,16,26,27,28,29,30] aimed to identify patients with a high chance to benefit from Sorafenib in terms of tumor response and OS, but none were based on a patient cohort as large as ours, which allowed us to assemble solid predictive models. In our model, the platelet count was inversely correlated with OS, a finding apparently in contrast with the classic paradigm, by which thrombocytopenia represents an index of advanced liver disease with clinically significant portal hypertension, and should therefore act as a negative prognostic factor [31]. Indeed, previous studies reported that a high platelet count is associated with a fast tumor growth rate [32,33], and Carr et al. recently elaborated a tumor aggressiveness score, named Liver Index, supporting the concept that patients with thrombocytosis have a more aggressive tumoral phenotype [34]. This association has been attributed to the role of the tumor microenvironment, as in vitro studies indicate that several platelet-derived growth factors (PDGF, EGF and serotonin) stimulate the expression of HCC cells and blunt the action of antiangiogenetic drugs such as Sorafenib and Regorafenib [35,36]. Taken together, these findings open the road to future studies targeting platelet-expressed growth factors as potential targets of new complementary therapies for HCC [37,38]. We also confirmed previous data [12,13,39,40,41] on the prognostic role of MTD regarding OS and tumoral progression. In particular, Carr et al. remarked on the presence of a correlation between MTD, AFP and AST levels with the survival of HCC patients [39]. As expected [8,30,42], high AFP levels (>200 ng/mL) heralded a poor OS, thus confirming that AFP-secreting HCCs have a more aggressive development, showing more frequently multifocality, portal vein invasion and hyperbilirubinemia [41]. Conversely, the ability of baseline AFP to predict tumor response to Sorafenib is controversial, while AFP changes over treatment appear to be more informative. In fact, an early AFP decrease after Sorafenib starting is considered a useful prognostic indicator, being correlated to a better prognosis than that observed in patients with stable or rising levels of this oncomarker [43,44,45,46]. As in our study we considered only pre-treatment AFP values, we could not address this issue. Intriguingly, low levels of hemoglobin were associated with poor survival. This may be explained by several factors, such the hypersplenism caused by portal hypertension leading to hemocateresis [31,47], or the myelosuppressive effect of Sorafenib, which can burden an already scarce state of general oxygenation. However, Sorafenib’s bone marrow toxicity is low [48,49] and has been reported in a few cases [50]. On the other hand, Finkelmeier et al. [51] found that patients with advanced HCC have lower hemoglobin levels in comparison with earlier stages, and this was related with survival.

As far as liver tests are concerned, we confirmed the independent negative prognostic meaning of bilirubin levels [52,53,54] as an index of baseline liver function which can be further hampered by features of tumor extension/aggressiveness, such as portal vein thrombosis, multifocality and higher AFP [39,53]. Even the prognostic role of AST levels is in line with previous reports [12,41,55,56,57,58], and may be explained considering that they express the necrotic “activity” of the liver disease upon which HCC ensued. Lastly, our study gives support to the pivotal prognostic role of the patient general status, expressed as ECOG PS, which is in fact a component of several prognostic models, such as the BCLC [59] and ITA.LI.CA [17] staging systems, as well as of the Sorafenib-dedicated prognostic models PROSASH-I [12] and SAP [14]. Our study has several limitations. First, we were not able to compute and test the predictive value of immune–inflammatory scores such as PLR [60], NLR [61] and SII [62] since the recording in the ITA.LI.CA database of variables forming these scores started in 2017. Second, we a priori excluded patients’ symptoms as potential prognosticators in order to provide models relying only on standardized parameters. Third, since our study focused on the development of a prognostic model based on pre-treatment variables, we did not include Sorafenib’s side effects, which have been proven to be a favorable prognostic factor [63,64,65,66]. Fourth, parameters such as time to tumor progression and progression-free survival were not evaluated due to the lack of relevant data in the ITA.LI.CA. database. However, in this respect, it is pertinent to note that the European Association for the Study of the Liver (EASL) guidelines consider OS the best goal for testing the efficacy of systemic therapies for HCC [3]. In addition, after building the model for the prediction of radiological progression according to mRECIST criteria, we found that its discriminatory ability was rather low and the decision curve analysis showed a very narrow range of net benefit, thus reducing its clinical value. On the other hand, the proposed model for OS survival showed a C-index value higher than those reported for many other prognostic models in a previous analysis [19]. In the current study, we decided to avoid the division of our cohort in a training set and an internal validation set to increase the power of our models. Lastly and more important, the prognostic models we proposed require external validations, and their performances should be compared to those of the already existing models. Indeed, external validation may provide data on the model’s reproducibility and generalizability. However, ideally, external validation should be performed in a separate study by different researchers to prevent adjustments of the model based on external validation results. All these issues represent the next steps of our research in this field. Nevertheless, our study also has several strengths. First, we reported data obtained from a very large cohort of prospectively enrolled patients by several academic and non-academic Italian centers, so that our analysis robustly validates the predictive ability of some factors found in small cohort of patients treated with Sorafenib. Furthermore, all prognostic parameters we propose are routinely measured in the field-practice work-up of HCC patients, so that their use does not add costs and complexity to this process. Moreover, deriving from real-world management, our data reliably reflect the results achievable in clinical practice with Sorafenib therapy in HCC patients, and the availability of nomograms based on routine parameters predicting the tumor response and patient survival may help to select patients who confidently will benefit from this treatment, ameliorating its suboptimal cost-effectiveness. After external validation of their prognostic accuracy in patients undergoing Sorafenib, our nomograms could be utilized to assess in patients deemed poor candidates for Sorafenib therapy the outcome of alternative first-line treatments, such as Lenvatinib or Atezolizumab plus Bevacivumab.

## 5. Conclusions

In conclusion, the present study proposes novel prognostic scores for patients undergoing Sorafenib therapy. The novelty introduced by these scores is a patient assessment based on common and cost-effective markers of patient’s general status, liver function and damage and HCC aggressiveness associated with the outcome of Sorafenib therapy in a real-life large cohort of HCC patients. Two predictive nomograms, helping clinicians in the therapeutic choice, were created. However, further studies aimed at validating the prognostic accuracy of these nomograms and comparing their performance with those of other models are needed.

## Figures and Tables

**Table 1:** Demographic, laboratory and clinical characteristics of the 1107 patients included in the study.

Characteristic	Number of Patients	Number (%) or Mean (SD)
Age at diagnosis of HCC (years)	1107	64.3 (13.0)
Sex (M)	1107	904 (81.7)
BMI	1107	25.07 (4.24)
Karnofsky score	1107	83.0 (13.7)
ECOG PS	1107	-
0	-	627 (56.7)
1	-	373 (33.7)
2	-	91 (8.2)
3	-	16 (1.4)
Liver disease etiology	1097	-
HCV	-	455 (41.5)
HBV	-	111 (10.1)
Alcohol	-	126 (11.5)
Metabolic	-	68 (6.2)
Other causes/multiple etiology	-	297 (27.1)
MELD score	1031	10 (3.22)
Child–Pugh class	1009	-
A	-	662 (65.6)
B	-	330 (32.7)
C	-	17 (1.7)
Esophageal varices	1107	436 (39.4)
HCC Features		
BCLC	1049	-
0-A	-	111 (10.2)
B	-	205 (18.9)
C	-	706 (65.1)
D	-	27 (2.5)
ITA.LI.CA. Prognostic Score	1107	-
1	-	54 (4.9)
2	-	113 (10.2)
3	-	566 (51.1)
4	-	374 (33.8)
Number of nodules	992	3 (2.4)
HCC Grading	312	-
1–2	-	163 (52.2)
3–4	-	149 (47.8)
Maximum tumor diameter (cm)	1107	4.70 (3.14)
Vascular invasion/thrombosis	1107	425 (38.4)
Absent	-	682 (61.6)
Portal vein	-	258 (23.3)
Peripheral	-	167 (15.1)
Metastases	1107	427 (38.6)
Extent >50% liver volume	996	131 (13.1)
Maximum tumor diameter (cm)	1107	4.70 (3.14)
Death	1107	882 (79.7)
Survival after initiation of Sorafenib (months) (median (IQR)) *	1107	10.1 (4.1; 20.2)
Time of Sorafenib administration (months) (median (IQR)) *	1085	4.1 (2.0; 12.2)
Response to Sorafenib (mRECIST)	780	-
Progression	-	517 (66.2)
Stable	-	166 (21.3)
Partial regression	-	77 (9.9)
Complete regression	-	20 (2.6)
Laboratory Tests		
Albumin (g/dL)	1056	3.54 (0.55)
Total bilirubin (mg/dL)	1056	1.33 (1.37)
INR	1021	1.19 (0.25)
ALT (multiple of UNL)	1107	1.63 (1.80)
AST (multiple of UNL)	1107	1.96 (2.42)
GGT (multiple of UNL)	1107	3.54 (3.72)
ALP (multiple of UNL)	1106	3.43 (3.77)
Alpha-fetoprotein (ng/mL)	1087	2404 (5955)
Creatinine (mg/dL)	1022	0.92 (0.45)
Na+ (mmol/L)	855	138.73 (4.2)
K+ (mmol/L)	851	4.30 (0.5)
Hemoglobin (g/dL)	962	12.70 (1.9)
Platelets (×10^9^/L)	979	144.9 (86.9)

*: expressed as median value (interquartile range); Abbreviations: HR, hazard ratio; CI, confidence intervals; ECOG PS, Eastern Cooperative Oncology Group (ECOG) Performance Status (PS); MELD, Model for End-stage Liver Disease; BCLC, Barcelona Clinic Liver Cancer; ITA.LI.CA., Italian Liver Cancer; cm, centimeters; HCC, hepatocellular carcinoma; INR, international normalized ratio; AST, aspartate aminotransferase; ALP, alkaline phosphatase; UNL, upper normal limit.

**Table 2:** Predictors of overall survival with statistical significance at the univariate and multivariate analysis.

Variable		Univariate		Multivariate	
		HR (95% CI)	*p*	HR (95% CI)	*p*
ECOG PS		1.287 (1.172; 1.414)	<0.001	1.284 (1.123; 1.460)	<0.001
MELD score		1.035 (1.017; 1.054)	<0.001	-	-
Child–Pugh class		1.357 (1.185; 1.556)	<0.001	-	-
BCLC stage		1.238 (1.132; 1.354)	<0.001	-	-
Esophageal varices		1.087 (1.010; 1.171)	0.026	-	-
Maximum tumor diameter (cm) *		1.077 (1.052; 1.102)	<0.001	1.100 (1.069; 1.133)	<0.001
Vascular invasion/thrombosis					
	Absent	Reference	-	-	-
	Portal vein	1.303 (1.109; 1.532)	0.001	-	-
	Peripheral	1.360 (1.125; 1.645)	0.001	-	-
HCC extension >50% liver volume		1.792 (1.460; 2.199)	<0.001	-	-
Albumin (g/dL) *		0.806 (0.710; 0.916)	0.001	-	-
Total bilirubin (mg/dL) *		1.082 (1.039; 1.127)	<0.001	1.119 (1.004; 1.246)	0.042
INR*		1.320 (1.032; 1.689)	0.027	-	-
AST (multiple of UNL) *		1.029 (1.002; 1.057)	0.035	1.032 (1.001; 1.065)	0.041
ALP (multiple of UNL) *		1.020 (1.003: 1.037)	0.023	-	-
Alpha-fetoprotein (≥200 ng/mL)		1.285 (1.136; 1.455)	<0.001	1.342 (1.113; 1.618)	0.002
Serum sodium (mmol/L) *		0.973 (0.955; 0.993)	0.007	-	-
Hemoglobin (g/dL) *		0.918 (0.885; 0.952)	<0.001	0.903 (0.860; 0.948)	<0.001
Platelets (×10^9^/L) *		1.002 (1.001; 1.002)	<0.001	1.002 (1.001; 1.003)	<0.001

*: per unit increase. Abbreviations: HR, hazard ratio; CI, confidence intervals; ECOG PS, Eastern Cooperative Oncology Group (ECOG) Performance Status (PS); MELD, Model for End-stage Liver Disease; BCLC, Barcelona Clinic Liver Cancer; cm, centimeters; HCC, hepatocellular carcinoma; INR, international normalized ratio; AST, aspartate aminotransferase; ALP, alkaline phosphatase; UNL, upper normal limit.

**Table 3:** Predictors of HCC progression (expressed according to mRECIST criteria) with statistical significance at the univariate and multivariate analysis.

Variable	Univariate		Multivariate	
	OR (95% CI)	*p*	OR (95% CI)	*p*
ECOG PS	1.270 (1.020; 1.581)	0.033	-	-
Maximum tumor diameter (cm) *	1.083 (1.025; 1.145)	0.005	1.068 (1.006; 1.134)	0.031
Albumin (g/dL) *	0.753 (0.570; 0.996)	0.047	-	-
Platelets (×10^9^/L) *	1.003 (1.001; 1.005)	0.001	1.003 (1.001; 1.005)	0.023

*: per unit increase. Abbreviations: OR, odds ratio; CI, confidence intervals; ECOG PS, Eastern Cooperative Oncology Group (ECOG) Performance Status (PS); cm, centimeters.

## Data Availability

Data is contained within the article or Supplementary Material.

## Supplementary Materials

The following are available online at https://www.mdpi.com/article/10.3390/cancers13112677/s1, Figure S1. Calibration plots regarding the 6-month overall survival calculated with the proposed nomogram, Figure S2. Decision curves of the proposed nomogram regarding the 6-month overall survival prediction, Figure S3. Calibration plots for the proposed nomogram regarding the HCC progression during Sorafenib treatment, Figure S4. Decision curves of the proposed nomogram regarding the HCC progression during Sorafenib treatment.
