# Supplementary material for: A Nomogram-Based Prognostic Model for Advanced Hepatocellular Carcinoma Patients Treated with Sorafenib: A Multicenter Study
Source: Cancers (Basel). 2021 May 29;13(11):2677. doi: 10.3390/cancers13112677 (PMC8199276; doi:10.3390/cancers13112677)
Supplement: Supplementary file 1 [file cancers-13-02677-s001.zip › cancers-1185927-supplementary.pdf]

Supplementary Materials: A Nomogram-Based Prognostic Model for Advanced Hepatocellular Carcinoma Patients Treated with Sorafenib: A Multicenter Study

Giovanni Marasco, Francesco Poggioli, Antonio Colecchia, Giuseppe Cabibbo, Filippo Pelizzaro, Edoardo Giovanni Giannini, Sara Marinelli, Gian Ludovico Rapaccini, Eugenio Caturelli, Mariella Di Marco, Elisabetta Biasini, Fabio Marra, Filomena Morisco, Francesco Giuseppe Foschi, Marco Zoli, Antonio Gasbarrini, Gianluca Svegliati Baroni, Alberto Masotto, Rodolfo Sacco, Giovanni Raimondo, Francesco Azzaroli, Andrea Mega, Gianpaolo Vidili, Maurizia Rossana Brunetto, Gerardo Nardone, Luigina Vanessa Alemanni, Elton Dajti, Federico Ravaioli, Davide Festi, Franco Trevisani, and the Italian Liver Cancer (ITA.LI.CA.) group

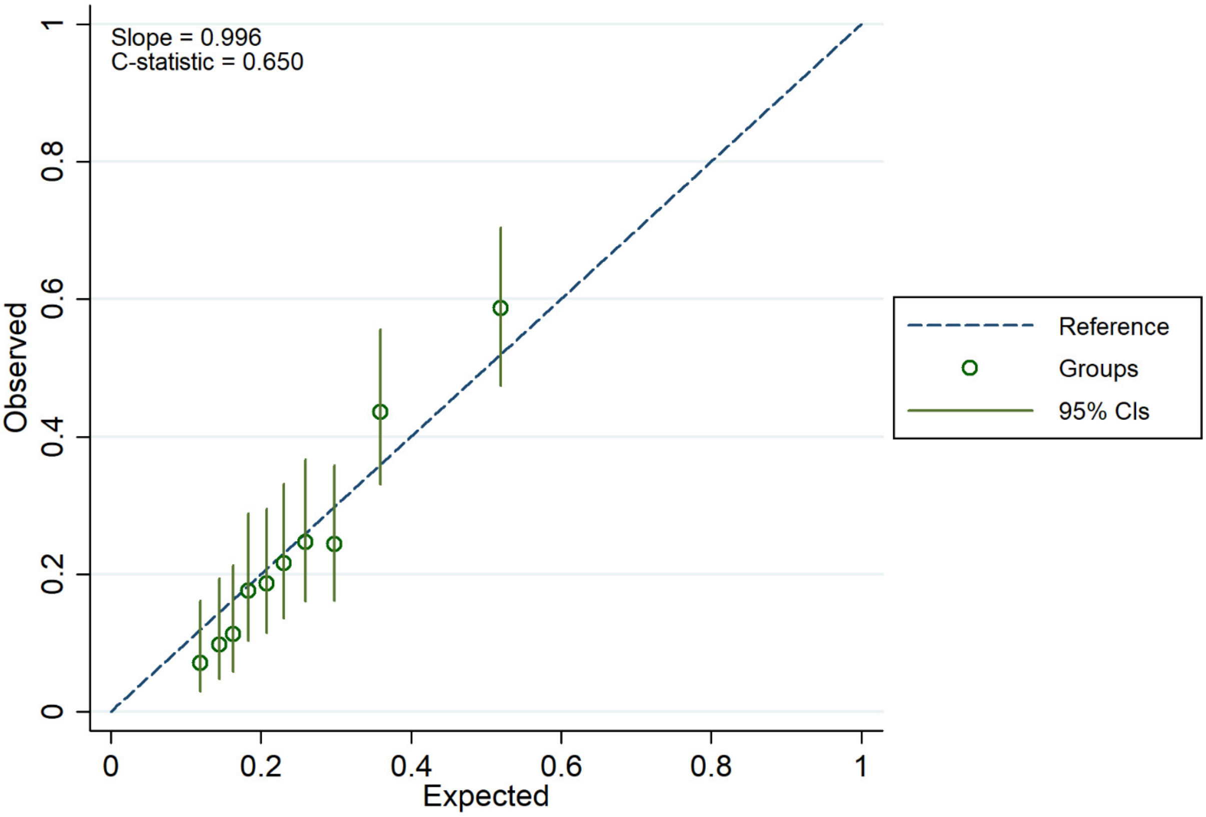

Figure S1. Calibration plots regarding the 6-month overall survival calculated with the proposed nomogram.

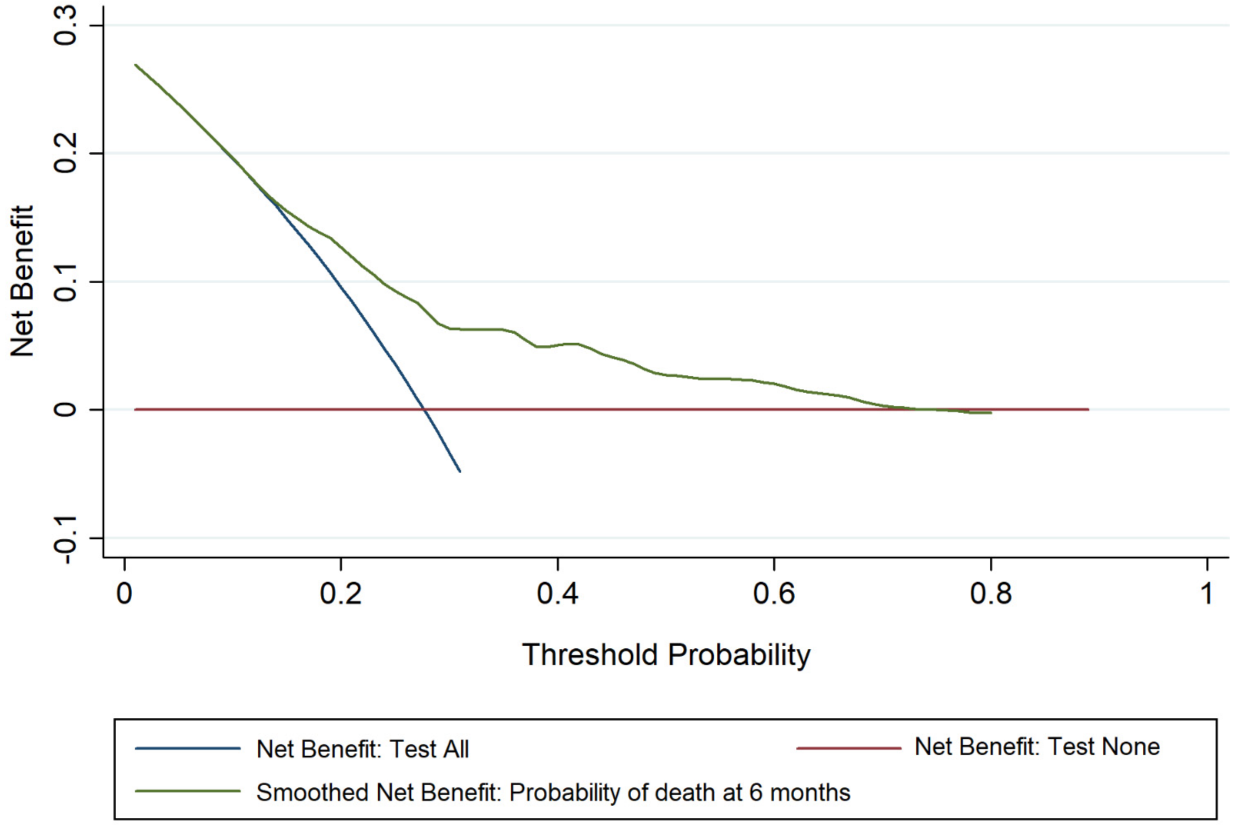

Figure S2. Decision curves of the proposed nomogram regarding the 6-month overall survival prediction.

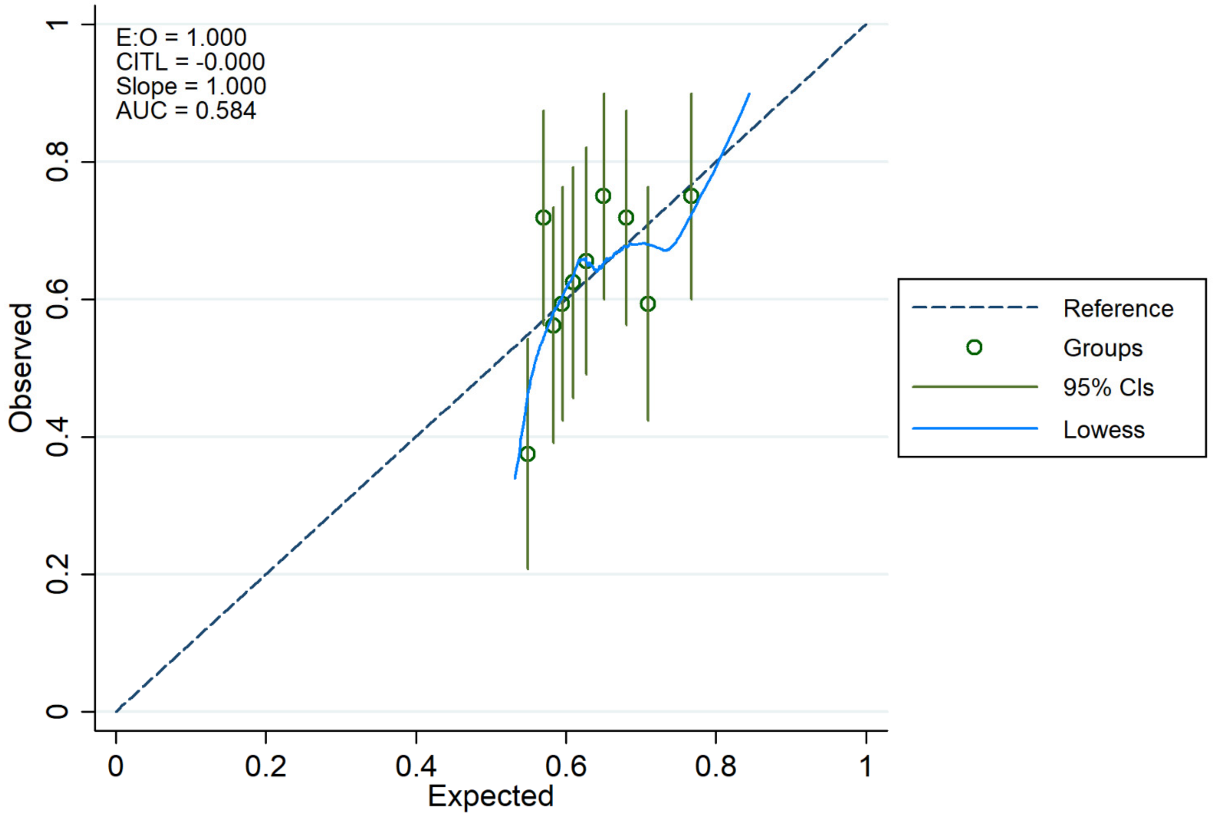

Figure S3. Calibration plots for the proposed nomogram regarding the HCC progression during Sorafenib treatment.

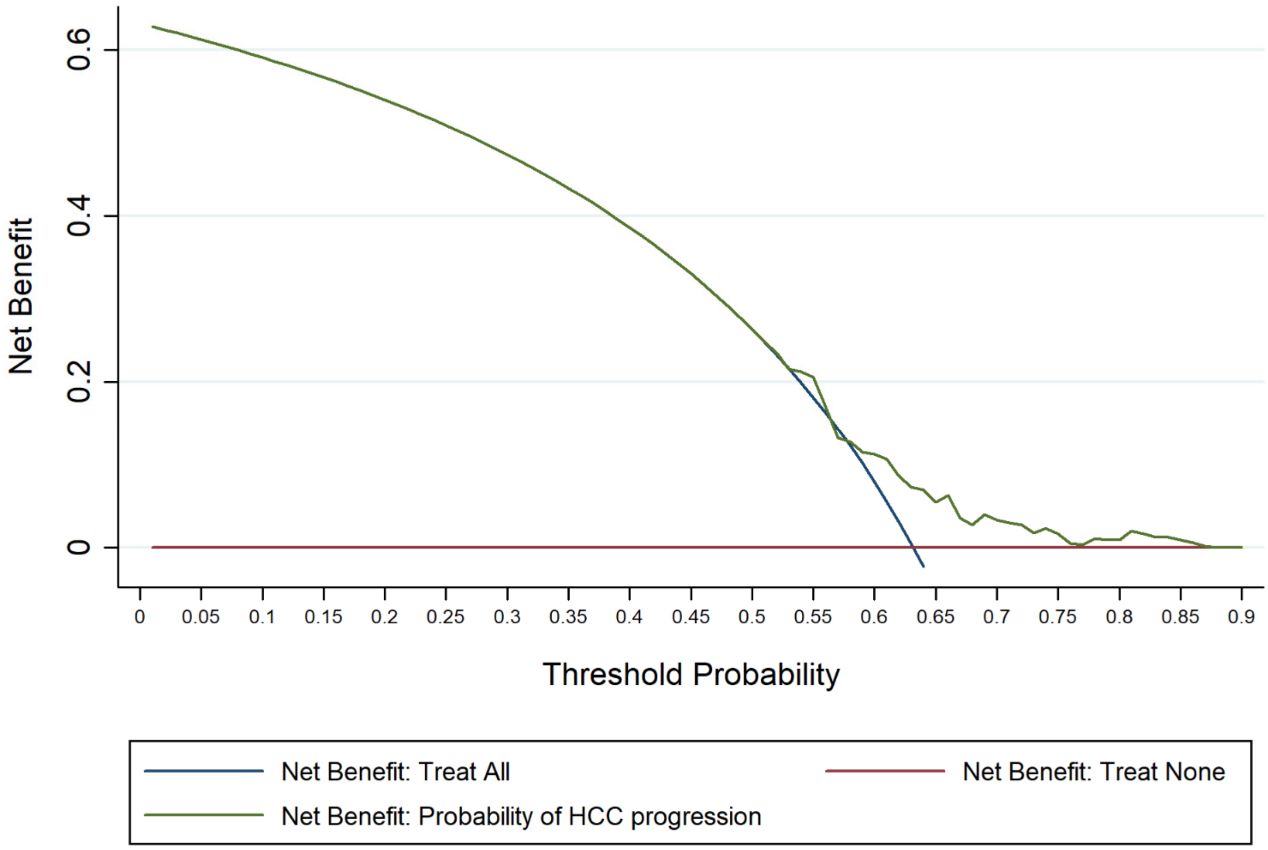

Figure S4. Decision curves of the proposed nomogram regarding the HCC progression during Sorafenib treatment.
